# The N-terminus of FILIA Forms an Atypical KH Domain with a Unique Extension Involved in Interaction with RNA

**DOI:** 10.1371/journal.pone.0030209

**Published:** 2012-01-19

**Authors:** Juke Wang, Mengyuan Xu, Kai Zhu, Lei Li, Xinqi Liu

**Affiliations:** 1 State Key Laboratory of Medicinal Chemical Biology, College of Life Sciences, Nankai University, Tianjin, China; 2 State Key Laboratory of Reproductive Biology, Institute of Zoology, Chinese Academy of Sciences, Beijing, China; National Institute for Medical Research, Medical Research Council, United Kingdom

## Abstract

FILIA is a member of the recently identified oocyte/embryo expressed gene family in eutherian mammals, which is characterized by containing an N-terminal atypical KH domain. Here we report the structure of the N-terminal fragment of FILIA (FILIA-N), which represents the first reported three-dimensional structure of a KH domain in the oocyte/embryo expressed gene family of proteins. The structure of FILIA-N revealed a unique N-terminal extension beyond the canonical KH region, which plays important roles in interaction with RNA. By co-incubation with the lysates of mice ovaries, FILIA and FILIA-N could sequester specific RNA components, supporting the critical roles of FILIA in regulation of RNA transcripts during mouse oogenesis and early embryogenesis.

## Introduction

RNA binding proteins (RBPs) play critical roles in germline and early embryonic development in model organisms by regulating RNA splicing, RNA subcellular localization, mRNA stability and translation [1], [2], [3], [4]. In mouse oocytes, RNA is transcribed and accumulated during oogenesis and most of the RNA is translated directly into proteins [5]. However, some RNA remains dormant and becomes activated later during oogenesis by carefully orchestrated polyadenylation [6]. The transcription becomes quiescent during meiotic maturation prior to ovulation and the majority of their polyadenylated RNA disappears after ovulation [7], [8]. Furthermore, most of the RNA in oocytes is degraded during the maternal-to-zygotic transition in the early stages of embryonic development following fertilization [9]. Therefore, RBPs have a significant physiological role during mouse oogenesis and early embryogenesis.

The hnRNP K homology (KH) domain was first identified in the human heterogeneous nuclear ribonucleoprotein K (hnRNP K) [10], [11]. The KH domain consists of approximately 70 amino acids and is well known for its RNA binding ability [2], [3], [12], [13], [14], but the consequences of this interaction are not completely understood. The KH domain usually acts in concert in a multiple-KH manner, and many KH-containing proteins, such as hnRNP K [10], FMR1 [15], and Nova [16], include more than one KH domains within a single molecule. However, some KH domain containing proteins possess only a single KH domain, such as Mer1p [17] and Sam68 [18]. Using *in vitro* assay systems, KH-containing proteins have been shown to typically bind poly-pyrimidine RNA [19]. It is implied that KH domain containing proteins play critical roles in gene expression by regulating pre-mRNA splicing, and by their involvement in polyadenylation, translation and RNA degradation. Accumulating evidence shows that KH-containing proteins function in various physiological processes, such as early embryonic development [20], neuron degeneration [21], [22], apoptosis [12], [23], and cancer development [24]. Currently, the direct link between the RNA binding activity of KH domains and their physiological consequences remain to be clarified.

Recently, we have identified a subcortical maternal complex (SCMC) in mouse oocytes and early embryos essential for cleavage-stage mouse embryogenesis [25]. The SCMC contains a core formed by FLOPED [20], MATER [26], TLE6, and FILIA. FILIA has been shown to directly interact with MATER, but not FLOPED and TLE6 [25]. The genes in the SCMC are transcribed and accumulate during oogenesis and degrade when the oocytes mature, but the protein components in SCMC exist until the blastocyst stage [25]. The lack of either FLOPED or MATER in oocytes does not affect folliculogenesis, ovulation, or fertilization, but leads to the failure of early embryos to complete cleavage stage development, resulting in a striking female sterile phenotype in these mutant mice [25], [26], [27]. However, *filia* ^tm/tm^ female mice are not completely sterile, and the early embryos in *filia* ^tm/tm^ female mice exhibit significant delays in their development. FILIA is important in regulation of mitotic kinase activity and spindle assembly checkpoint activation, and contributes to chromosome stability [28], although the molecular basis for the relationship between its special localization and its unique function is not clear.

*Filia* is located in Chromosome 9 in mouse, and is encoded by a single-copy gene [29]. *Filia* is transcribed as two types of transcripts with respective lengths of 1.2 k and 1.6 k base pairs that co-exist in oocytes, but only the former transcript is translated into a functional protein with 346 residues. The FILIA protein is unique in primary sequence without any clearly identifiable domains. The C-terminus of FILIA is composed of 10 tandem repeats with each repeat comprising 23 residues. This arrangement of repeats was identified for the first time in FILIA and no homology to other known proteins has been observed [29], [30]. *Filia* has been classified into a new oocyte/embryo expressed gene family in eutherian mammals, along with *khdc1*, *dppa5*, and *floped* [31]. Proteins in this gene family are characterized by an atypical N-terminal KH domain and their sequence varies greatly beyond the KH domain. Compared to other canonical KH domains, a conserved N-terminal extension is identified prior to the KH domain in these proteins, although their function remains unknown.

In order to study the structural basis for its function, we expressed and crystallized the N-terminal domain of FILIA ranging from 1 to 124 amino acids (named FILIA-N hereafter). The structure of FILIA-N shows several features unique to FILIA and the probable function of the N-terminal extension prior to the KH domain was studied with regard to RNA binding. Furthermore, FILIA-N forms a stable dimer in both crystals and solution with high affinity, which is distinct from canonical KH domains.

## Results

### 1. Overall view of the FILIA-N structure

**Figure 1:**
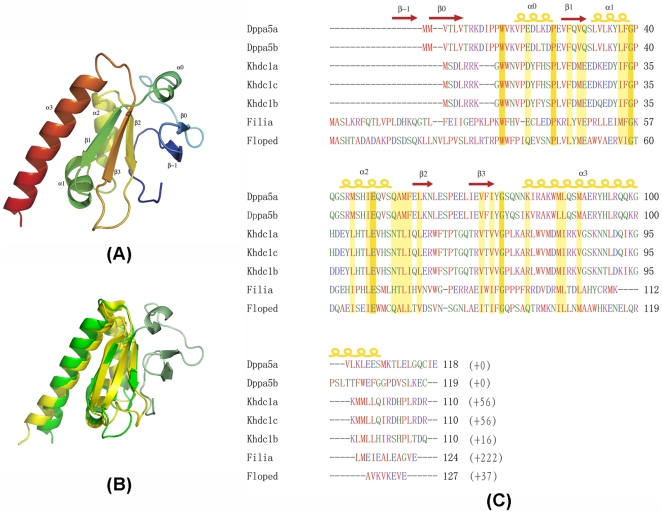
The structure of FILIA-N. (A) Overall structure of FILIA-N. Molecule is shown colored from blue at the N-terminus to red at the C-terminus, and secondary structure elements are labeled. (B) Superimposition of FILIA-N (monomer1) and Nova2-KH3. FILIA-N is colored green from residues 40–117, and pale green from residues 2–39; Nova2-KH3 is shown in yellow. (C) Sequence alignment for members of the oocyte/embryo expressed gene family by ClustalW [60]. All sequences are from mouse, and only the N-terminal KH region is shown. The number following the sequence in parentheses refers to the number of remaining residues in the variable C-terminus of the protein. Residues are colored according to their characteristics, i.e., red for hydrophobic, green for polar, blue for negatively charged and purple for positively charged. Invariant residues are shown with brown background, and conserved residues are shown with yellow background. Secondary structure elements are labeled on top of the alignment, as in Fig. 1A.

The crystal structure of FILIA-N (1–124 AA) was solved by the single wavelength anomalous diffraction (SAD) method (see Materials and Methods for details). FILIA-N crystallized with two molecules in an asymmetric unit forming a stable dimer. The monomer structure of FILIA-N was used to search for similar known 3-dimensional structures in the Protein Data Bank by DALI [32]. The hit yielding the highest score (Z score 6.9) was the KH3 domain of Nova2 (PDB code 1EC6) [33], which represents a canonical type I KH domain. From residues 40–118, FILIA-N forms a classical KH module with a β-α-α-β-β-α topology (Fig. 1A). The root mean square deviation (RMSD) between the two KH domain structures is 1.85 Å based on superposition of Cα atoms from FILIA residues 40–118 and Nova2-KH3 residues 6–83 (Fig. 1B). A salient structural feature of FILIA-N was the presence of an N-terminal extension prior to the FILIA KH region (Fig. 1A). Starting with an unstructured stretch, this loop-β-β-α substructure capped one surface of the KH domain. In particular, the two anti-parallel β-stands (β-1 and β0, Fig. 1A) are positioned almost perpendicular to the β2-β3 sheet within the KH domain, thus extending the canonical RNA binding area of the KH domain as deduced from the Nova2-KH3/RNA complex (PDB code 1EC6). Interestingly, the beginning stretch of around 12 residues of FILIA-N lies in a groove formed by the KH domain and the N-terminal extension, contributing to the overall stability of the whole molecule. The N-terminus of other oocyte/embryo-expressed proteins, such as Dppa5 and Khdc1, possess a much shorter N-terminal extension of around 20 residues (Fig. 1C). Compared with Nova2-KH3, it is evident that the three β-strands constituting the core of the molecule are well conserved in conformation in FILIA-N, but three α-helices, namely α1, α2, and α3, were tilted a little relative to their equivalents in Nova2-KH3. Significant differences were found in a non-conserved loop region between β2 and β3. This loop was 4 residues shorter in FILIA-N than in Nova2-KH3, shrinking laterally in space and leaving more room for the N-terminal residues of the extension region (Fig. 1C).

On the surface of Nova2-KH3, a negatively charged groove was employed for interaction with RNA. By superposition of FILIA-N with the Nova2-KH3/RNA complex (PDB code 1EC6), a similar negatively charged patch on the surface of FILIA-N, involving the N-terminal extension, was also identified. Interestingly, a positively charged area adjacent to this negatively charged patch formed mainly by β0 and α0 was also observed, the function of which is unknown.

### 2. Unique dimerization of FILIA-N

**Figure 2:**
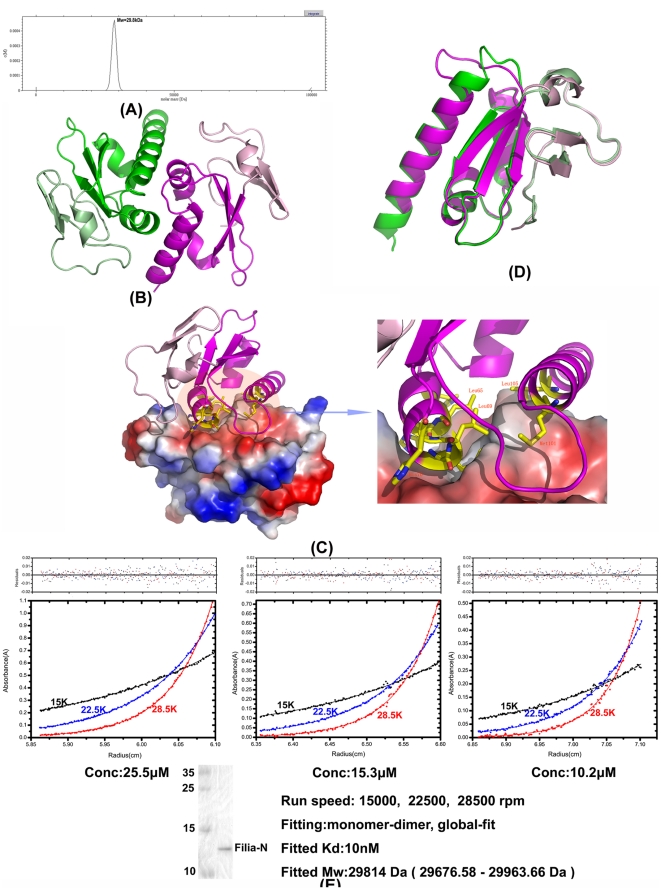
Dimerization of FILIA-N. (A) Sedimentation velocity analysis of FILIA-N. The peak corresponds to a molecular mass of 29 KDa, indicating a dimer in solution. (B) Dimer in an asymmetric unit. Monomer1 is colored green from residues 40–117, and pale green from residues 2–39; monomer2 is colored magenta from residues 40–114, and light pink from residues 4–39 AA. (C) Interaction surface within dimer. Monomer1 of FILIA-N is shown represented by electrostatic surface potential, while monomer2 is shown in ribbon representation. The residues involved in the interaction between monomer1 and monomer2 are shown in stick representation and depicted in detail on the right picture. (D) Superposition of monomer1 and monomer2 of FILIA-N, colored as in Fig. 2(B). (E) Protein concentrations plotted versus radius for an AUC equilibrium experiment. 25.5, 15.3 and 10.2 µ*M* FILIA-N were spun at 15,000, 22,500 and 28,500 rpm at 4°C. The solid line showed a fit of the data to a model of a dimer with a molecular weight of 29,814 (rmsd of 0.005). The molecular weight of the monomer calculated from its sequence is 14,625 Da. Residuals are shown at the top of the plot. A SDS-PAGE gel of FILIA-N used in this experiment is also shown.

KH domains are well known for their oligomerization ability, and it is postulated that these molecules mediate their roles in a concerted multi-KH manner accompanying RNA binding, though in most cases the interactions between KH domains are weak and unstable. In contrast, FILIA-N was confirmed to form a stable dimer in solution by analytical ultra-centrifugation (Fig. 2A), and this observation was further supported by our crystal structure. In our orthorhombic crystals, two FILIA-N molecules in one asymmetric unit formed a stable dimer (Fig. 2B). Nova2-KH3 also formed dimers [21] although, despite the overall high similarity between the individual KH domains of FILIA-N and Nova2-KH3, the two molecules exhibited a significantly different mode of interaction in their dimerization interface. From the top view along the non-crystallographic 2-fold symmetry axis, one monomer in FILIA-N was rotated by about 30° relative to another monomer when compared against the Novo2-KH3 dimer, leading to a much more stable interaction surface. The total buried area in the dimer of FILIA-N was approximately 1920 Å^2^ compared to 930 Å^2^ for the Nova2-KH3 dimer. This data lead us to speculate that FILIA functions as a dimer *in vivo*. On the dimerization surface, most of the interactions involve the helices α2 and α3. Hydrophobic interactions contribute most of the stabilizing forces, including residues Met68, Met101, Leu65, Leu69, and Leu105. Three histidines, His61, His64, and His70, form hydrogen bonds with a neighboring molecule at the edge of the buried surface. The loop between β3 and α3 from monomer2 protrudes into a shallow pocket on the surface of monomer1 (monomer 1 and 2 are named according to their chain name), with the benzyl ring of Phe94 playing an anchoring role (Fig. 2C). While considering the high flexibility of this region, we doubt this interaction plays a critical role in stabilization of the dimer. The N-terminal extension is located outside of the interaction interface and contributes to a surface for possible RNA binding (Fig. 2C).

Comparison of two monomers within one dimer showed several differences. With the exception of the different orientation of the first 5 residues at the N-terminus, the loop between β3 and α3 is the most significant divergence between the two monomers. In monomer 2, this loop protruded out towards monomer 1. In monomer 1, however, this loop was reorganized and most of the loop region participated in the formation of α3, forming a continuous α-helix along with the following residues. This loop region showed large flexibility, as evidenced by high temperature factors and poor electron density, and possibly exists in varying conformations depending on the surrounding environment. Another substantial difference between the two monomers was located in α0 and β1, which correspond to the beginning of the canonical KH domain (Fig. 2D). Furthermore, the residues in α3 that were assigned from electron density were different in the two monomers, indicating that the length of α3 could vary between different molecules provided the stability of the KH domain was maintained. The RMSD between the two monomers was 0.65 Å based on the Cα atoms of all residues (Fig. 2D).

The homo-dimerization of KH domains has long been proposed, but solid data to support the existence of this kind of homo-dimer has been tenuous [34]. We identified a stable KH domain homo-dimer in our FILIA-N crystals and demonstrated its existence in solution by sedimentation velocity measurements (Fig. 2A). In order to confirm this result, a sedimentation equilibrium measurement was performed to analyze the precise affinity of this homo-dimer. Purified FILIA-N with various concentrations was detected under different centrifugal forces to measure the dissociation constant of this dimer. A measured molecular mass of 29814 Da was obtained with a *K*d of 10 nM, indicating a stable dimer with high affinity (Fig. 2E). This, to the best of our knowledge, is the first KH domain homo-dimer with such high affinity to be identified to date. In contrast, the Nova2-KH3 region is shown to exist as a monomer in solution by sedimentation velocity (Fig S1).

### 3. The unique feature of the N-terminal extension in FILIA-N

**Figure 3:**
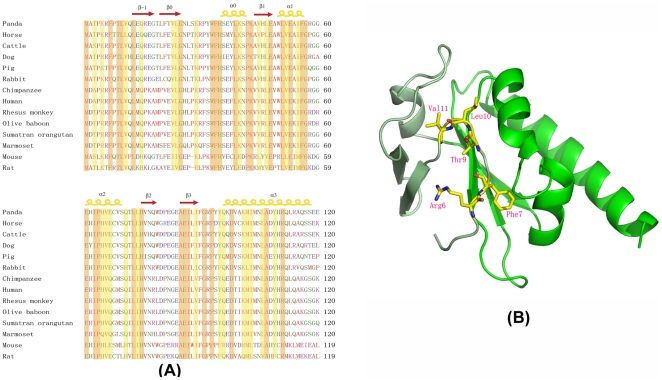
Features of the N-terminal extension. (A) Sequence alignment of FILIA-N from different mammals. Residues are colored and labeled as in Fig. 1(C). (B) Interaction of the N-terminal fragment (AA 2–12) with other parts of FILIA-N. The dominant and conserved residues within different mammals are shown in stick representation and labeled.

The N-terminal 39 residues prior to the KH region form a unique substructure in FILIA-N. This substructure is composed of a loop at the beginning (residues 2-12), followed by two anti-parallel β-strands (β-1 and β0) and an α-helix (α0). Interestingly, in the newly identified oocyte/embryo specific gene family which includes *dppa5*, *khdc1*, *filia* and *floped*, the cognate proteins Dppa5 and Khdc1 possess a short extension around 20 residues, while FILIA and FLOPED had extensions of approximately 40 residues at their N-termini. A sequence alignment shows that all of these proteins are conserved in their KH regions and the preceding α0 helix, but not in other regions, indicating these oocyte/embryo specific proteins possess both a conserved KH domain and an α-helical (α0) extension. Both FILIA and FLOPED have a much longer N-terminal extension compared to Dppa5 and Khdc1, but no sequence similarity could be identified between these two proteins prior to the α0 helix, reminiscent of their divergent roles in embryogenesis based on *filia* and *floped* knockout mice [25], [28]. To investigate the significance of this N-terminal extension, we performed a Blast search for orthologous proteins from other mammals against mouse FILIA. Interestingly, this N-terminal extension is conserved in different species with comparable sequence identity to each other, as with the following conserved KH region, suggesting the similar loop-β-β-α extension also exists in other mammals (Fig. 3A). This result underlines the importance of the N-terminal extension in the function of FILIA. In contrast, a similar Blast search for FLOPED suggested that the N-terminus preceding α0 in FLOPED from different species is quite divergent in both length and sequence, indicating a non-conserved and/or even less critical N-terminus for FLOPED. Taken together, the loop-β-β-α extension is a unique substructure in FILIA and might have emerged later during gene evolution within the oocyte/embryo specific gene family.

The first 12 residues in FILIA-N form a loop situated between two substructures formed by the N-terminal extension and the typical KH region, effectively bridging the whole molecule together. By sequence alignment of FILIA from different species, three invariant residues, i.e., Phe7, Thr9 and Leu10, were identified. Mutations of these residues to alanine reduced the solubility of these proteins when over-expressed in *E. coli* or *sf9* insect cells (data not shown), implying the importance of these residues in maintaining the overall structure of FILIA-N. In our FILIA-N crystal structure, Phe7 protrudes into a hydrophobic pocket surrounded by Leu51, Phe55, Ile62, Val75 and Val77, contributing to the stabilization of the local conformation. Thr9 forms a stable hydrogen bond with Glu66, and the conservation of Glu or Gln at position 66 in various species reiterates the importance of this interaction. Leu10 and the following conserved Val11 are also important for structural stabilization by hydrophobic interaction with surrounding residues (Fig. 3B). By measuring the secondary structure of these mutants with circular dichroism, all proteins had similar spectra to the wild-type protein, with a minor difference around the valley at 225 nm, implying these mutants are correctly folded but exhibit some differences on local conformation (Fig S2).

### 4. The interaction between FILIA and RNA stretch from poly-C and poly-U

**Figure 4:**
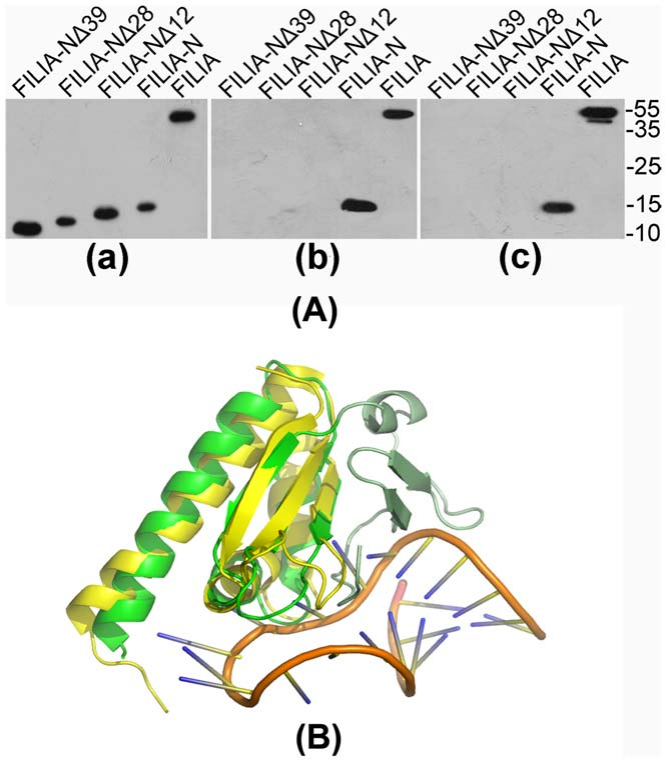
Interaction of FILIA with RNA. (A) The binding of FILIA or FILIA-N with poly-C or poly-U RNA. In panel (a), purified recombinant proteins (FILIA, FILIA-N, FILIA-NΔ12, FILIA-NΔ28 and FILIA-NΔ39) were detected by anti-6xHis antibody. Proteins are labeled on top and molecular mass of marker bands are shown on right. In panel (b), purified recombinant proteins were sequestered by poly-C ribonucleotide homopolymers and detected by anti-6xHis antibody. In panel (c), purified recombinant proteins were sequestered by poly-U ribonucleotide homopolymers and detected by anti-6xHis antibody. (B) Nova2-KH3/RNA complex (PDB code 1EC6) was superimposed onto FILIA-N. Proteins are colored as in Fig. 1(C), and the RNA molecule is shown in ribbon representation.

The KH domain has long been recognized to bind RNA, but the consequences of this binding remain unclear. To date, the exact RNA sequence that binds to FILIA is unknown, and thus poly-C and poly-U segments were used here as substitutes for authentic RNA to test the interaction between FILIA and RNA. As residues 40–124 of FILIA-N form the KH domain, and residues 1–39 form an extension ahead of KH, we made a series of truncated FILIA-N constructs and examined their ability to bind RNA. Both the full-length FILIA and FILIA-N proteins bound RNA with similar binding ability. Surprisingly, although the FILIA 40–124 (FILIA-NΔ39) protein lacking the N-terminal extension still possessed a KH domain, it completely lost the ability to bind RNA. Other constructs with varying lengths of sequence at the N-terminus, i.e., FILIA 13–124 (FILIA-NΔ12) lacking only an unstructured stretch at the N-terminus, and FILIA 29–124 (FILIA-NΔ28) retaining the conserved α0, also lost their ability to bind RNA (Fig. 4A). These results support the indispensable roles of the N-terminal extension ahead of the canonical KH domain in RNA binding. The N-terminal extension forms a new layer of β-sheet on top of the original 3-strand β-sheet formed by β1-β2-β3, constituting a new β-sheet-β-sheet-α-helix three-layer conformation. The peptide from residues 2–12 is located in a groove formed by the β-1-β0-sheet and the β1-β2-β3 sheet, bridging the N-terminal extension and canonical KH region, thus contributing towards the stability of the whole molecule.

The folding of these mutants, i.e. FILIA 13–124, FILIA 29–124, FILIA 40–124, were detected by circular dichroism to exclude the possibility of misfolding and/or aggregation leading to the loss of RNA binding. All of these mutants shared similar spectra to the wild-type protein, indicating the correct folding of these proteins. Further denaturing by high temperature of 85°C without/with 4 M guanidine distorted the spectrum either partially or completely, which further strengthens our conclusion (Fig S3).

The authentic RNA substrate that binds to FILIA is unknown to date. Taking advantage of the availability of the structure of the Nova2-KH3/RNA complex (PDB code 1EC6), we superimposed FILIA-N onto the Nova2-KH3/RNA complex. Clearly the sequence and/or conformation of the RNA fragment from the Nova2-KH3/RNA complex were not suitable for binding to FILIA-N. Most of the conflicts arose from residues 2–12, which clashed with the RNA derived from the Nova2-KH3/RNA complex. The other clash arose from the variable loop between β2 and β3 (Fig. 4B). On the RNA side, most of the clashes were derived from U12-C16, the core segment for Nova2-KH3-RNA recognition. Due to the flexibility of RNA, it is difficult to speculate further on the detailed aspects of the interaction between FILIA-N and its authentic RNA sequence. The structure of FILIA in complex with authentic RNA substrate(s) is required to elucidate the details of this interaction.

### 5. FILIA binds intrinsic RNA in ovaries

**Figure 5:**
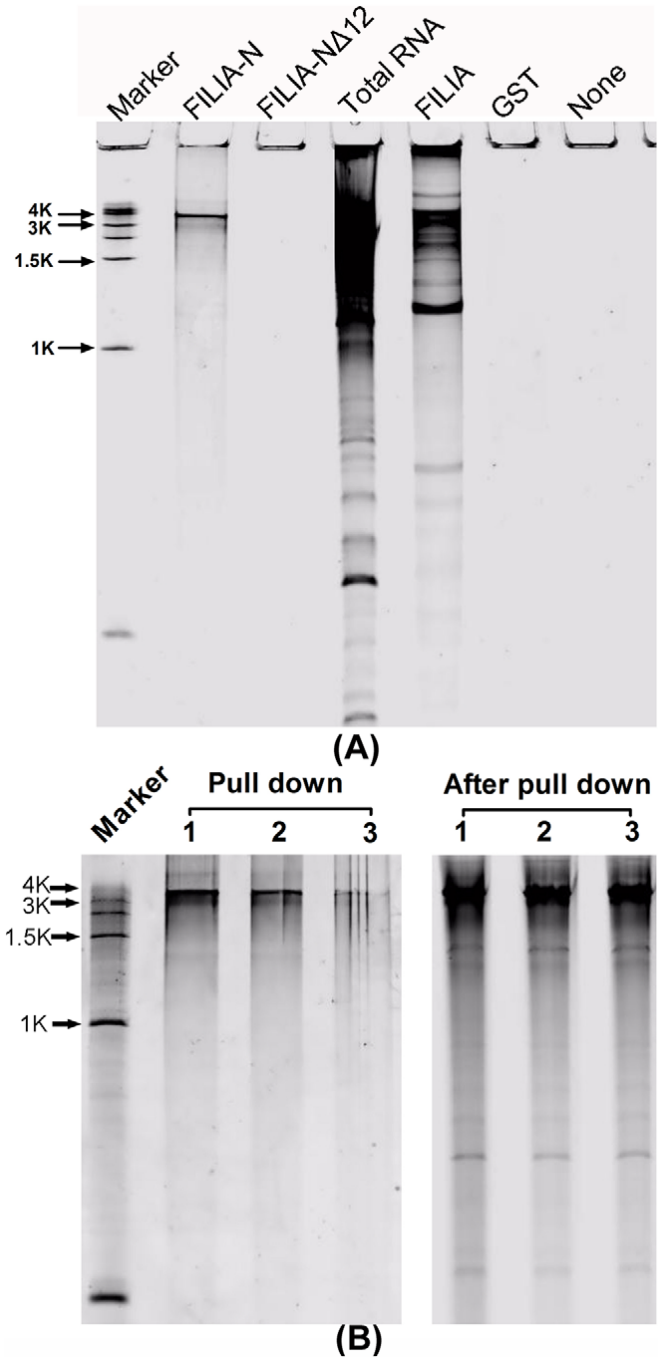
Intrinsic RNA pulled down by FILIA-N, FILIA-NΔ12 and FILIA. (A). FILIA-N and FILIA pull-down intrinsic RNA. Total RNA was purified from mice ovaries and incubated with FILIA-N, FILIA-NΔ12, FILIA, GST and Ni-NTA beads. The pull-down RNA and total RNA were separated by urea denatured PAGE and stained with SYBR Green II. The results were scanned with FLA 7000. (B). Repeating pull-down experiments. Pull-down lane 1 was RNA pulled down by FILIA-N from total ovarian RNA; Pull-down lane 2 was RNA pulled down by FILIA-N from the first residual RNA; Pull-down lane 3 was RNA pulled down by FILIA-N from the second residual RNA; After pull-down lane 1 was the first residual RNA; After pull-down lane 2 was the second residual RNA; After pull-down lane 3 was the third residual RNA.

As FILIA has the ability to bind RNA *in vitro*, we tried to determine if FILIA could bind intrinsic RNA *in vivo*. FILIA was specifically expressed in oocytes and early embryos. Due to the scarcity of intrinsic FILIA in oocytes, we used recombinant FILIA proteins purified from *E. coli* to pull-down the total RNA from mouse ovaries (see Materials and Methods for details). FILIA-N, but not FILIA 13–124 or GST, clearly pulled down a specific RNA band sized between 3 Kbs and 4 Kbs from ovarian RNA, although full-length FILIA pulled down several bands including the specific RNA band from total ovarian RNA (Fig. 5A). The specificity of FILIA-N binding RNA was demonstrated by repeating pull-down experiments in which the residual RNA after pull-down was further incubated with FILIA-N. The bound RNA in repeated pull-down experiments was significantly decreased, although there were no significant differences in the residual RNA (Fig. 5B).

## Discussion

FILIA is a member of a recently identified oocyte/embryo expressed family whose members are characterized by an atypical KH domain. This protein family, including Khdc1a, Khdc1b, Khdc1c, Dppa5a, Dppa5b, Dppa5c, FILIA (Ecat1) and FLOPED (OOEP/MOEP19), are specifically expressed by oocytes and play important roles in oogenesis, folliculogenesis, or early embryo development in eutherian mammals [31]. Khdc1a/Ndg1 was originally described as a downstream target of Nur77, a nuclear orphan steroid receptor in T-cells, and may be involved in apoptosis [31], [35]. Khdc1b and Khdc1c are splicing isoforms of Khdc1a and may play important roles during mouse oogenesis and early embryonic development [31], [36]. Dppa5/ESG1/PH34/ECAT2 was first isolated as a gene down-regulated during the differentiation of embryonic carcinoma cells [37], [38] and then as a gene expressed specifically in mouse ES cells, germline cells [37] and pre-implantation embryos [39]. Although Dppa5 is dispensable in ES cells and mice according to gene mutant studies [37], Dppa5 was shown to interact with many mRNA including *cdc25a*, *cdc42*, *ezh2*, *nfyc* and *nr5a2* and regulate the expression of these genes [37], [40]. FLOPED was originally identified as MOEP19 (mouse oocyte and early embryo protein 19) and was shown to bind oligonucleotides using an *in vitro* assay system [20]. In the present study, we have demonstrated that the crystal structure of the N-terminus of FILIA is similar to the Nova2-KH3 domain and shown that FILIA binds polynucleotides and endogenous RNA *in vitro*. Furthermore, the identification of a unique N-terminal extension preceding the canonical KH region in FILIA-N structurally distinguishes FILIA from other KH domain containing proteins. Overall, the results described above indicate this atypical KH domain in this oocyte/embryo expressed gene family binds RNA like other canonical KH-domain proteins, but might have unique consequences that are restricted to oocytes and early embryonic development.

Compared to other canonical KH-containing proteins, such as Nova-1, Nova-2 [16], FBP [41], [42] and Vigilin [43], [44], [45], these proteins possess only one KH domain in their N-terminus and the precise roles of these proteins are unclear. The postulated KH domain of FILIA is distinct from other canonical KH proteins. First, the primary sequence is not conserved with other canonical KH domains. The KH domain of FILIA therefore cannot be predicted solely from its amino acid sequence. Second, the N-terminal 39 amino acids of FILIA form a substructure attached to the KH domain, which plays a critical role in RNA binding. The N-terminal extension sequence is unique among the oocyte/embryo expressed gene family, but the length of the extension varies between different proteins. Khdc1a, Khdc1b, Khdc1c and Dppa5 share a very short N-terminal extension varying between 15–20 residues, while FLOPED and FILIA possess a long N-terminal extension of up to 40 residues [31]. The latter are both components of a recently identified subcortical maternal complex (SCMC) [25]. Interestingly, although the N-terminal extension is shorter in Khdc1 and Dppa5, their additional N-terminal residues can be precisely aligned to the N-terminal α0 region of FILIA. From the sequence alignment, we can conclude that at least helix α0 is also conserved in this oocyte/embryo-expressed gene family besides the KH domain.

As previously described, the KH domain generally functions in a multiple-KH manner in KH domain containing proteins. In the current study, we demonstrated that FILIA-N forms a stable dimer in solution and in the crystal structure. Furthermore, our previous study showed that FILIA and FLOPED, which both contain KH domains, co-exist in the SCMC [25]. Due to their similar expression profiles and their versatile binding with RNA via KH domains, we speculate that FILIA and FLOPED function, either as a homo-dimer or in a multiple-KH manner together in the SCMC *in vivo*, in RNA degradation during oocyte maturation or early embryogenesis. Further studies to identify the specific RNA substrate of this novel atypical KH domain or SCMC will help to elucidate the mechanism of RNA degradation in oogenesis and early embryogenesis in mammals.

The homodimerization of KH domains and the relationship to their function has been proposed for a long time, but conclusive evidence for the existence of homo-dimers formed by KH domains is lacking. Most of the buried dimerization area identified in KH domain crystal structures was less than 1200 Å^2^, a required area for a stable dimer suggested by Janin et al. [46]. Recently two KH domain dimers with large buried surface areas were reported, i.e., PCBP2 KH1 (1890 Å^2^, PDB code 2AXY) and hFMRP KH1-KH2 (2100 Å^2^, PDB code 2QND). Nevertheless, in PCBP2, no solution data to support the existence of a stable dimer is available [47]; and in hFMRP, analytical ultracentrifugation measurements clearly showed that the protein is monomeric in solution [48]. A monomer-dimer equilibrium with 10–20% dimer of Nova1 KH3 in solution was also suggested by Ramos et al. [49], but a low dissociation constant in the millimolar range was roughly estimated and no quantitative data was provided by further experiments. Taken together, these data imply that the possible KH domain homo-dimers but with large uncertainty. In this manuscript, we described the structure of FILIA-N, which forms a stable dimer in both crystals and in solution with a dissociation constant of about 10 nM, and we analyzed the dimer interface in detail. To the best of our knowledge, this is the first case of an isolated KH domain forming high affinity dimers using only elements of the KH fold. The STAR/GSG-containing proteins (signal transduction and activation of RNA/GRP33, Sam68, GLD-1) also form homo-dimers, but their dimerization predominantly involves the QUA1 region preceding the KH domain [50], and the latter behaves as a monomer in the absence of the QUA1 motif [51].

Structural extensions are present in some KH domains. For example, QUA1 and QUA2 extensions prior to and following the KH domain in STAR/GSG-containing proteins, such as sam68, QK1 and GLD-1, have been identified for a long time [18]. Nevertheless, unlike the N-terminal extension described here, QUA1 is composed of a coiled-coil that dimerizes perpendicularly to each other, and play largely autonomous roles with little direct interaction with the subsequent KH domain [50]. Furthermore, the unavailability of a structure for the full STAR/GSG domain encompassing both QUA1 and KH domains to date prevents the comparison of these substructures to the loop-β-β-α extension in FILIA-N in the context of the KH region. A β0-turn0 element prior to the canonical KH region was also found in the structure of KSRP KH1, in which β0 interacts with β1 in the KH domain in an anti-parallel manner [52]. However, this extension does not bury hydrophobic side chains and its deletion has little effect on protein stability. Both N-terminal extensions in STAR/GSG and KSRP are not involved in RNA binding directly. Therefore, these extensions are completely different from that of FILIA-N as described here, in which the N-terminal extension of around 40 residues forms a tight contact with the canonical KH region, contributing to both structural stability and the functional role of this protein. This unique extension, which is inseparable from the canonical KH region, defines a new extended KH domain that is presumed to function in a distinct manner from regular KH domain-containing proteins in mammalian oogenesis and early embryogenesis.

Considering the significance of the interaction between oocyte-specific factors and RNA transcripts, and the importance of this new eutherian oocyte/embryo expressed gene family in mammalian oogenesis and early embryogenesis, our results not only reveal the unique features of these oocyte/embryo specific proteins in RNA binding, but also provide a structural basis for possible intervention of abnormal embryonic development such as habitual abortion.

## Materials and Methods

### Ethics Statement

All research involving animals in this study follow the guidelines and byelaws governing experiments on animals, and have been approved by the Ethics and Experimental Animal Committee of the Institute of Zoology, Chinese Academy of Sciences. The Animal Research Committee does not issue a number to a specific study. But each study requires the permit to use animals from the Committee, and this research was under the supervision of the Committee for all procedures. The animal facility at Institute of Zoology gets licensing from the experimental animal committee of Beijing city and the animal handling staff (including each post-doc and doctor student) must be trained before using animals. The mice were killed by cervical dislocation. The only procedure performed on the dead animals is the collection of oocytes from the oviduct. Oocytes were collected according to procedures described previously [25] and were used for one time to extract total RNA. No cell lines were established and no oocytes were kept for further use.

### 1. Cloning, expression and purification

The gene encoding full-length filia was cloned from total RNA from mouse ovary cells. The primers 5′-TACTTCCAATCCAATGCCATGGCCTCTCTGAAG-3′ (forward) and 5′-TTATCCACTTCCAATGCTACTCAACTCCAGCCTC-3′ (reverse) were designed to amplify the sequence coding for amino acids 1–124 of FILIA (FILIA-N). The target sequence was cloned into the pET30-TEV/LIC Vector (Novagen) downstream of a 6×His tag via the ligation independent cloning method.

The plasmid carrying the target gene was transformed into *Escherichia coli* BL21 (DE3) strain. Cells were grown in LB Broth medium and induced with 0.3 m*M* isopropyl-β-D-thiogalactopyranoside (IPTG) for protein expression. Cell lysis was performed by sonication on ice in Ni-NTA resin binding buffer (50 m*M* Tris, 500 m*M* NaCl, 4 m*M* imidazole, pH 8.0). The lysate was clarified by centrifugation and supernatant was loaded onto a Ni-NTA resin column (Qiagen). The 6×His-FILIA-N protein was eluted in elution buffer (binding buffer with 500 m*M* imidazole). After being concentrated in an Amicon Ultra filter (Millipore), the protein was dissolved into low salt buffer (50 m*M* Tris, 50 m*M* NaCl, pH 8.0). Afterwards, the 6×His tagged protein was digested by TEV protease at 289 K for 24 h. Following removal of the 6×His tag, the protein was purified by HiTrap Q HP column (GE Healthcare) and then loaded onto a HiLoad 16/60 Superdex-200 size-exclusion column (GE Healthcare) for further purification. A sharp peak corresponding to the target protein was pooled and concentrated to 15 mg ml^−1^ for further crystal screening.

For expression of a seleno-methionyl derivative protein, the pET30-FILIA-N vector was transformed into methionine auxotroph strain B834 (DE3). Cells were then cultured with M9 minimal medium supplemented with 50 µ*g* ml^−1^ L-methionine to an absorption value of 1.0 at OD_600_. After L-methionine depletion, 50 µ*g* ml^−1^ seleno-methionine was provided and 0.2 m*M* IPTG was used to induce protein expression. The isolation and purification procedures were the same as for the native protein.

Human Nova2-KH3 fragment (residues 406–492) was cloned, expressed and purified by a similar procedure as described above. The protein lacking the N-terminal 6×His tag was chromatographically purified prior to analytical ultracentrifugation experiments.

### 2. Crystallization and data collection

FILIA-N crystals were grown by the hanging-drop vapor diffusion method at 277 K. A 2 µl droplet of protein solution (15 mg ml^−1^) mixed with an equal amount of mother liquor was equilibrated against 500 µl reservoir solution (1.4 *M* ammonium sulfate, 0.1 *M* Tris 8.2, 12% v/v Glycerol) to yield FILIA-N crystals suitable for data collection. The seleno-methionyl derivative protein was crystallized by 0.2 *M* ammonium formate, 16% w/v PEG3350.

Data collection from native crystals was performed at 100 K using a wavelength of 0.9798 Å at the Photon Factory (KEK), Tsukuba, Japan. Data from seleno-methionyl derivative crystals were collected at the Shanghai Synchrotron Radiation Facility (SSRF), Shanghai, China. For data collection under cryogenic conditions, crystals were soaked for a few seconds in a mixed solution of mother liquor with 20% (v/v) glycerol. Crystals were then mounted on the beam line in a nylon loop and flash-cooled in a liquid-nitrogen stream at 100 K. Native data sets were collected by rotating 180° with an increment of 1° per frame. For the derivative crystal, a single dataset was collected at the peak wavelength of 0.9795 Å over 180°. All data sets were processed using the HKL2000 package [53]. Data collection and processing results are summarized in Table 1.

**Table 1:** X-ray crystallographic data and refinement statistics for FILIA-N.

Crystals	Native	Se-derivative (peak)
**Data collection**		
Space group	*P2_1_2_1_2_1_*	*P2_1_2_1_2_1_*
Unit cell dimensions		
a, b, c (Å)	38.30, 73.96, 89.96	56.54, 59.16, 89.62
α, β, γ (°)	90, 90, 90	90, 90, 90
Molecules per ASUΦ	2	2
Resolution (Å)*	2.2(2.32-2.2)	2.8(3.04-2.8)
Completeness (%)*	98.0(95.7)	98.7(92.5)
Redundancy*	5.4(2.1)	5.9(2.3)
No. of unique reflection*	14029(801)	9324(624)
I/σ*	16.2(2.7)	17.4(2.6)
R_sym_ * †	9.2(42.4)	7.4(29.9)
Figure of merit		0.5
**Refinement statistics**		
Resolution (Å)	2.2	
No. of reflections	13955	
R_work_/R_free_ (%)‡ §	21.08/27.53	
No. of atoms		
Protein	1896	
Water	102	
B-factors (Å^2^)		
Protein	42.33	
Water	44.01	
R.m.s. deviations		
Bond length (Å)	0.007	
Bond angle (°)	1.187	
**Ramachandran analysis**		
Most favored (%)	89.6	
Additional allowed (%)	9.9	
Generously allowed (%)	0.5	
Disallowed (%)	0	

Φ ASU = asymmetric unit.

*Values in parentheses are for the highest resolution shell.

† Rsym  =  Σ|I−<I>|/Σ<I>, where I is the observed intensity, and <I> is the average intensity of multiple observations of symmetry related reflections.

‡ R  =  Σhkl||Fobs|−|Fcalc||/Σhkl|Fobs|.

§ Rfree is calculated from 5% of the reflections excluded from refinement.

### 3. Structure determination

Due to the high quality of the data, we successfully located the positions of 9 out of 12 selenium atoms in an asymmetric unit from the peak data set, and the preliminary model was readily built following single-wavelength anomalous diffraction (SAD) [54], [55] phasing using the Phenix package [56]. The model built using the experimental SAD phases was used as a starting model for molecular replacement against high-resolution native data. The Phenix refinement program (phenix.refine) was used in refinement. Simulated annealing, positional refinement and B-factor refinement were used in multiple rounds. Non-crystallographic symmetry restraints were not applied during refinement. Ordered water molecules were added to the structure in the last round of refinement. Refinement statistics are summarized in Table 1.

### 4. Analytical ultracentrifugation

Sedimentation velocity (SV) and sedimentation equilibrium (SE) experiments were performed using a Beckman/Coulter XL-I analytical ultracentrifuge with double-sector or six-channel centerpieces and sapphirine windows. An additional protein purification step on a HiLoad 16/60 Superdex 200 gel-filtration column in 50 m*M* Tris pH 8.0, 200 m*M* NaCl and 1 m*M* TCEP was performed before the experiments. SV experiments were conducted at 60,000 r.p.m and 4°C using absorbance detection and double-sector cells loaded with approximately 43 µ*M* for FILIA-N and 84 µ*M* for Navo2-KH3. For the SE experiments, data were collected at 4°C and 15,000, 22,500 and 28,500 r.p.m with 25.5, 15.3 and 10.2 µ*M* FILIA-N respectively. The buffer composition (density and viscosity) and protein partial specific volume (V-bar) were obtained using the program SEDNTERP (http://www.rasmb.bbri.org/). The SV and SE data were analyzed using the programs SEDFIT and SEDPHAT [57], [58].

### 5. Circular dichroism spectroscopy

This protocol was adapted from a previously published method [59]. Briefly, CD spectra were recorded in a 1 cm path-length cuvette using BioLogic MOS 450 (Science Instruments, Inc.) at 20°C and 85°C. Protein samples were diluted in CD buffer (50 m*M* phosphate buffer pH 8.0, 200 m*M* NaCl) or denaturation buffer (CD buffer with 4 *M* guanidine hydrochloride) to a final concentration of 10 µ*M*. For each sample spectrum recorded, a buffer blank was subtracted from the raw signal.

### 6. Interaction of FILIA with poly-C and poly-U RNA

Full length FILIA, FILIA-N lacking the N-terminal 12 residues (FILIA-NΔ12), FILIA-N lacking the N-terminal 28 residues (FILIA-NΔ28) and FILIA-N lacking the N-terminal 39 residues (FILIA-NΔ39) were cloned and expressed using the same protocol for wild-type FILIA-N described above. Purified proteins were incubated with poly-C and poly-U agarose beads (SIGMA) in a binding buffer (10 m*M* Tris, 100 m*M* NaCl, 2.5 m*M* MgCl_2_, 0.5% Triton X-100 pH 7.4). After thorough washing, the beads were boiled in loading buffer (300 m*M* Tris-HCl, pH 8.0, 10% SDS, 20 m*M* EDTA, 25% β-mercaptoethanol, 0.1% bromophenol blue, 50% glycerol) and loaded onto an SDS-PAGE (polyacrylamide gel electrophoresis) gel. After running for 1 hour at 180 voltages, samples were transferred to PVDF membrane and blotted by anti-6xHis antibody (Santa Cruz). HRP-labeled secondary antibody (SIGMA) was used to produce chemiluminescent signals.

### 7. FILIA interaction with intrinsic RNA from mouse ovaries

Ovary total RNA was purified from homogenized 6 week old CD1 mouse ovaries with TRIzol (Invitrogen) and digested by DNase (Promega). After phenol-chloroform extraction, the precipitated RNA was dissolved in interaction buffer (50 m*M* NaH_2_PO_4_, 50 m*M* NaCl, 20 m*M* Imidazole, 0.005% Tween20, pH 8.0) and incubated with Ni-NTA Magnetic Agarose Beads (Qiagen) which were pre-sequestered with 6xHis tagged proteins (FILIA, FILIA-N, FILIA-NΔ12 and GST) at room temperature for 2 hours. After washing 6 times with interaction buffer, the bound RNA was eluted by high salt buffer (50 m*M* NaH_2_PO_4_, 1 *M* NaCl, 20 m*M* Imidazole). The pulled down RNA was further purified by co-precipitation with glycogen (0.5 ug ul^−1^) and loaded onto 5% urea denatured PAGE. After running for 1.25 hours at 200 V, the gel was stained with SYBR Green II for 10 minutes and scanned with FLA 7000 (Fujifilm). Repeating pull-down experiments were performed by re-incubating residual RNA with FILIA-N sequestered beads and then eluting and analyzing as above.

## Supporting Information

Figure S1 **Sedimentation velocity analysis of Nova2-KH3.** The peak corresponded to a molecular mass of 11 KD, indicating a dominant monomeric form in solution (predicted 9749 dalton). An unknown component of 2.63 KD co-purified with Nova2-KH3 impeded our trial of sedimentation equilibrium analysis of this protein.
(TIF)

Figure S2 **Circular dichroism analysis of N-terminal mutants of FILIA-N.** (a) Circular dichroism result of FILIA-N, FILIA-N F7A, FILIA-N T9A and FILIA-N L10A at 20°C. Four proteins were colored as blue, green, cyan and red, respectively. (b) Circular dichroism result of different conditions of FILIA-N. Results of 20°C, 85°C, and 85°C with 4 M guanidine hydrochloride were colored by red, green and blue, respectively. (c), (d), (e) Circular dichroism result of different conditions of FILIA-N F7A, FILIA-N T9A, and FILIA-N L10A. Results under various conditions were colored as in (b).
(TIF)

Figure S3 **Circular dichroism analysis of N-terminal truncations of FILIA-N.** (a) Circular dichroism result of FILIA-N, FILIA-NΔ12, FILIA-NΔ28 and FILIA-NΔ39 at 20°C. Four proteins were colored by blue, green, cyan and red, respectively. (b) Circular dichroism result of different conditions of FILIA-N. Results at 20°C, 85°C, and 85°C with 4 M guanidine hydrochloride were colored by red, green and blue, respectively. (c), (d), (e) Circular dichroism result of different conditions of FILIA-NΔ12, FILIA-NΔ28, and FILIA-NΔ39. Results under various conditions were colored as in (b).
(TIF)
