# Supplementary figures and images for: The N-terminus of FILIA Forms an Atypical KH Domain with a Unique Extension Involved in Interaction with RNA
Source: PLoS One. 2012 Jan 19;7(1):e30209. doi: 10.1371/journal.pone.0030209 (PMC3261892; doi:10.1371/journal.pone.0030209)

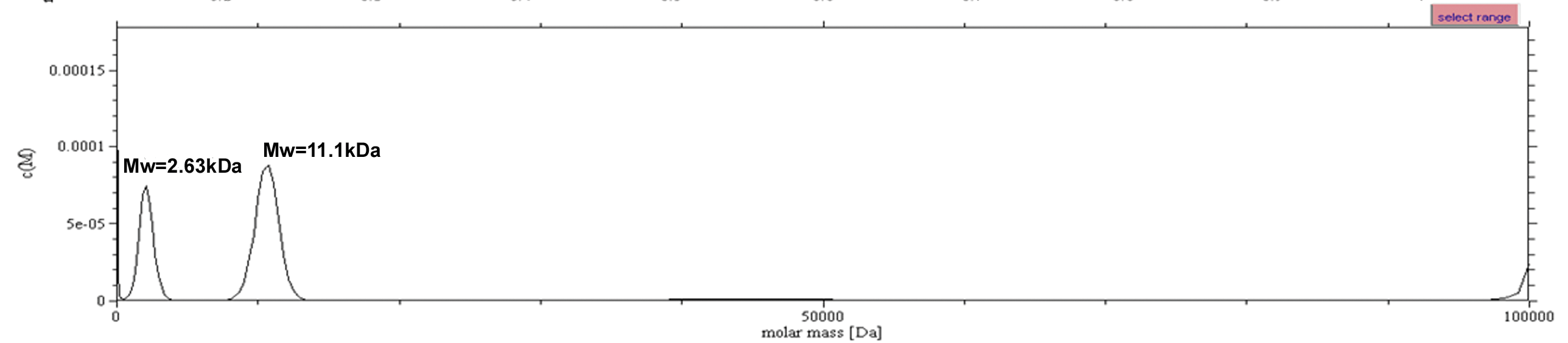

Supplement: Figure S1 — Sedimentation velocity analysis of Nova2-KH3. The peak corresponded to a molecular mass of 11 KD, indicating a dominant monomeric form in solution (predicted 9749 dalton). An unknown component of 2.63 KD co-purified with Nova2-KH3 impeded our trial of sedimentation equilibrium analysis of this protein. (TIF) [file pone.0030209.s001.tif]

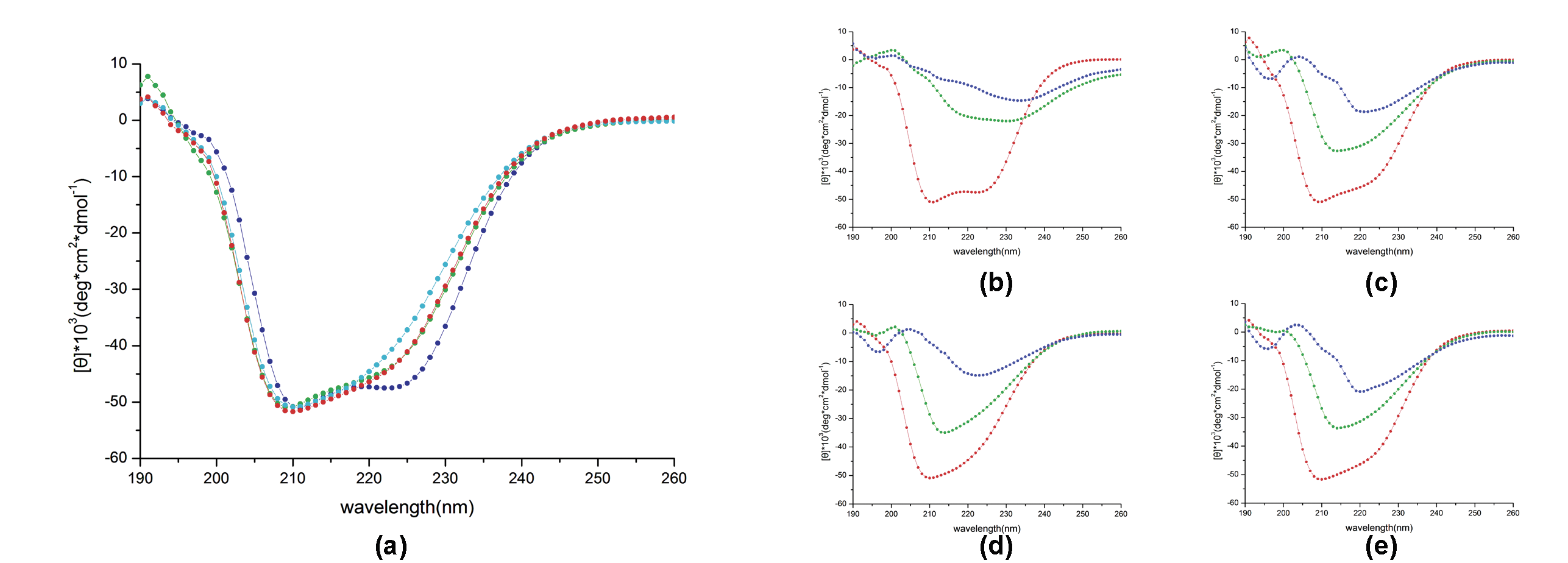

Supplement: Figure S2 — Circular dichroism analysis of N-terminal mutants of FILIA-N. (a) Circular dichroism result of FILIA-N, FILIA-N F7A, FILIA-N T9A and FILIA-N L10A at 20°C. Four proteins were colored as blue, green, cyan and red, respectively. (b) Circular dichroism result of different conditions of FILIA-N. Results of 20°C, 85°C, and 85°C with 4 M guanidine hydrochloride were colored by red, green and blue, respectively. (c), (d), (e) Circular dichroism result of different conditions of FILIA-N F7A, FILIA-N T9A, and FILIA-N L10A. Results under various conditions were colored as in (b). (TIF) [file pone.0030209.s002.tif]

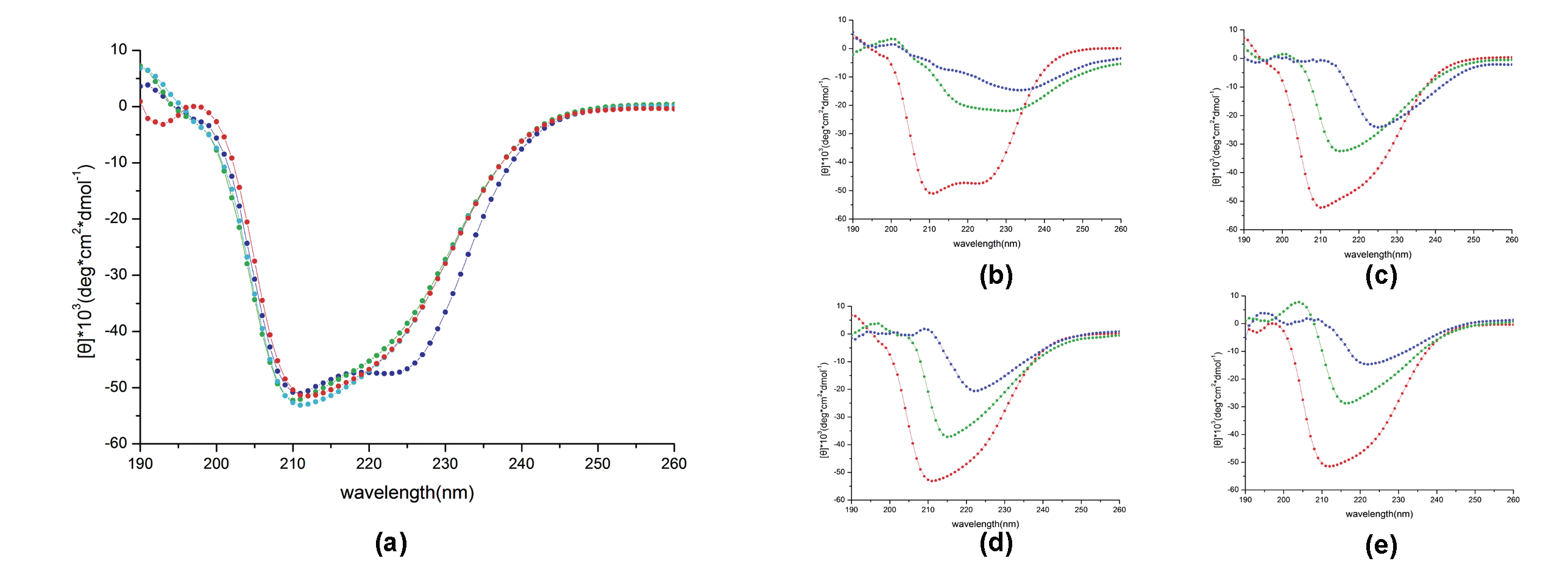

Supplement: Figure S3 — Circular dichroism analysis of N-terminal truncations of FILIA-N. (a) Circular dichroism result of FILIA-N, FILIA-NΔ12, FILIA-NΔ28 and FILIA-NΔ39 at 20°C. Four proteins were colored by blue, green, cyan and red, respectively. (b) Circular dichroism result of different conditions of FILIA-N. Results at 20°C, 85°C, and 85°C with 4 M guanidine hydrochloride were colored by red, green and blue, respectively. (c), (d), (e) Circular dichroism result of different conditions of FILIA-NΔ12, FILIA-NΔ28, and FILIA-NΔ39. Results under various conditions were colored as in (b). (TIF) [file pone.0030209.s003.tif]
